# Impact of alcohol drinking on total cancer risk: data from a large-scale population-based cohort study in Japan

**DOI:** 10.1038/sj.bjc.6602277

**Published:** 2004-12-14

**Authors:** M Inoue, S Tsugane

**Affiliations:** 1 Epidemiology and Prevention Division, Research Center for Cancer Prevention and Screening, National Cancer Center, 5-1-1 Tsukiji, Chuo-ku, Tokyo 104-0045, Japan

**Keywords:** alcohol drinking, cancer risk, cohort study, population-based

## Abstract

We conducted a cohort study of alcohol consumption and total cancer incidence and mortality in 73 281 subjects (35 007 men and 38 274 women) aged 40–59 years old at baseline over a 10-year follow-up period. During 1990–2001, a total of 3403 cases of newly diagnosed cancer and 1208 cancer deaths were identified. In men, the lowest risk of developing cancer was observed among occasional drinkers, and a linear positive association with increased ethanol intake was noted (hazard ratio 1.18 for 1–149 g per week, 1.17 for 150–299 g per week, 1.43 for 300–449 g per week, 1.61 for ⩾450 g per week, *P* for trend <0.001). The positive relation was similar for cancer incidence and mortality, but was more striking among current smokers and alcohol-related cancers. Relatively few women were regular drinkers. Our results suggest that increased ethanol intake linearly elevates the risk of cancer, and that nearly 13% of cancers among males in this study were due to heavy drinking (⩾300 g per week of ethanol), to which smoking substantially contributed. The simultaneous reduction of smoking is therefore important for reducing the effect of alcohol on cancer risk.

In Japan, both alcohol consumption and the proportion of heavy drinkers have been increasing for decades (The Editorial Board of the Cancer Statistics in Japan, 2003), and alcohol drinking has been recognised as an important and preventable public health problem. A quantitative estimation of the effects of alcohol drinking in a target population, with regard to not only specific cancers but also total cancers, is important in formulating public health policies. However, evidence of the association between alcohol and total cancer risk mainly concerns Western populations and cancer mortality (Blackwelder *et al*, 1980; Blot, 1992; Doll *et al*, 1994; Fuchs *et al*, 1995; Camargo *et al*, 1997; Renaud *et al*, 1998; Berberian *et al*, 1994; Gaziano *et al*, 2000; Bagnardi *et al*, 2001; Theobald *et al*, 2001). Little has been reported for Japanese or other ethnic groups (Kono *et al*, 1986; Yuan *et al*, 1997, Tsugane *et al*, 1999).

As the epidemiological background, types of beverage regularly consumed and genetic polymorphisms for alcohol-related enzymes in these ethnic groups differ from those in Western populations, we have conducted a cohort analysis of the question using a large-scale population-based prospective study with a 10-year follow-up period.

## METHODS

### Study population and baseline survey

The Japan Public Health Center-based prospective Study (JPHC Study) was launched in 1990 for Cohort I and in 1993 for Cohort II. Cohort I covered five prefectural public health center (PHC) areas and Cohort II covered six PHC areas. The details of the study design have been described elsewhere (Tsugane and Sobue, 2001). The study protocol was approved by the institutional review board of the National Cancer Center, Japan. In the present analysis, two PHC areas were excluded since different definitions of the study population had been applied.

The study population was defined as all registered Japanese inhabitants in the nine PHC areas aged 40–59 years at the start of each baseline survey. Initially, 96 616 subjects were identified but after excluding 178 subjects with non-Japanese nationality (*n*=45), late reports of emigration occurring before the start of the follow-up period (*n*=131), and incorrect birth date (*n*=2), a population-based cohort of 96 438 subjects (48 240 men and 48 198 women) was established.

A baseline self-administered questionnaire survey on various lifestyle factors was conducted in 1990 for Cohort I and in 1993–1994 for Cohort II, with a response rate of 81%. After excluding subjects with a self-reported serious illness (cancer, cerebrovascular disease, myocardial infarction, or chronic liver disease) and those without details of alcohol status, 73 281 subjects (35 007 men and 38 274 women) remained for analysis.

Information on alcohol intake was obtained in terms of frequency and the amount using validated questions (Otani *et al*, 2003; Tsubono *et al*, 2003). The average frequency was reported in six categories for cohort I: <1 day per month, 1–3 days per month, 1–2 days per week, 3–4 days per week, 5–6 days per week, and everyday. Subjects consuming alcoholic beverages at least once a week were also asked about the types of drinks consumed and the average consumption. Subjects in cohort II were also asked about their drinking status, never-, ex-, or current drinkers. Ex- and current drinkers provided information on the average frequency, the types of drinks consumed and average daily consumption. The average frequency of consumption was divided into the following categories, to each of which a score was assigned: 1.5 for 1–2 days per week, 3.5 for 3–4 days per week, and 6 for 5–6 days per week and everyday in the cohort I questionnaire, and 1.5 for 1–2 days per week, 3.5 for 3–4 days per week, and 6 for almost everyday in the cohort II questionnaire. The amount of ethanol by type of beverage was calculated as follows: 180 ml of sake (rice wine) was regarded as 23 g of ethanol, 180 ml of shochu or awamori (white spirits) as 36 g, 633 ml of beer as 23 g, 30 ml of whiskey or brandy as 10 g, and 60 ml wine as 6 g. Finally, the weekly ethanol intake was estimated by multiplying the amount by the score. In the present analysis, alcohol drinking was classified into six categories: nondrinkers (<1 day per month), occasional drinkers (1–3 days per week), and four categories of regular drinkers (1–149, 150–299, 300–449, and ⩾450 g per week).

### Follow-up and analysis

Subjects were followed from the baseline survey until December 31, 2001. Residence status, including survival, was confirmed annually through the residential registry kept in each municipality of the areas where the study subjects resided. Among the study subjects, 6.5% moved away and 0.06% were lost to follow-up during the study period. Information on the cause of each death was supplemented by checking against death certificate files with permission, and the cause of death was defined according to the International Classification of Disease, 10th Version (ICD-10) (WHO, 1990).

Cancers were identified by active patient notification from the local major hospitals in the study area and approved data linkage with the population-based cancer registries. Death certificates were used as a supplementary information source. Cases were coded using the International Classification of Diseases for Oncology, Third Edition (ICD-O-3) (WHO, 2000). In 2.2% of cancer cases, information was available only from death certificates (DCO). The earliest date of diagnosis was used in cases with multiple primary cancers at different times.

Person-years were accrued from baseline survey until the following end points: for cancer incidence – the date of occurrence of cancer, the date of emigration from the study area, the date of death, or the end of the study period, whichever came first; for total cancer deaths, the date of emigration from the study area, the date of death, or the end of the study period, whichever came first. Persons who were lost to follow-up were censored at the last confirmed date of presence in the study area.

The study outcomes were defined as newly occurring cancers of any site and all cancer deaths during the study period. Hazard ratios (HR) and their 95% confidence intervals (95% CI) were used to describe the relative risk cancer deaths associated with the alcohol categories at baseline (nondrinkers, occasional drinkers, 1–149 g of ethanol per week, 150–299, 300–449, and ⩾450 g per week), with ‘occasional drinkers’ representing the reference category. In men, stratified analyses were further conducted to evaluate whether alcohol effects and cancer risk varied with smoking status. Interaction terms were generated by multiplying the ordinal smoking categories by ordinal alcohol drinking categories. The HRs were further estimated separately for alcohol-related cancers, namely cancer of the oral cavity, pharynx, larynx, oesophagus, and liver (IARC, 1988; WHO, 2003) (ICD-0-3: C00-C10, C12-C15, C22, C32), and for cancers not considered to be alcohol-related. The Cox proportional hazards model was used to control for such potential confounding factors as age at baseline (continuous), study area (nine PHC areas), smoking status (pack-years (0, 1–19, 20–29, 30–39, ⩾40)), green vegetable intake (⩽3–4 times per week, everyday), and leisure-time physical activity (⩽1–3 times per month, ⩾1–2 times per week). These variables are either known or suspected risk factors for cancer or had been found to be associated with cancer risk in previous studies (Tsugane *et al*, 1999; Hara *et al*, 2002).

To express the impact of alcohol drinking on the risk of overall cancer, the population-attributable fraction (PAF) (%) was estimated as pd × (HR−1/HR), where pd is the proportion of cases exposed to the risk factors. This formula is considered more valid than the popular formula Pe × (RR−1)/(Pe × (RR−1)+1), where Pe is the proportion of the source population exposed to the risk factor, when a confounding variable exists (Rockhill *et al*, 1998). Confidence intervals (95%) for the adjusted PAF were estimated using the formula of Greenland (1999). Stata version 8 special edition software (Stata Corporation, 2003) was used to perform the statistical analyses.

## RESULTS

During the 721 302.5 person-years of follow-up (average: 9.8 years) for the 73 281 subjects (35 007 men and 38 274 women), a total of 3403 newly diagnosed cancers (1904 men and 1499 women) and 1208 cancer deaths (758 men and 450 women) were included in the analyses. With regard to cancer incidence, gastric cancer was the commonest cancer in men (*n*=533, 28.0%), followed by colon (*n*=281) and lung cancers (*n*=230); in women, breast cancer was commonest (*n*=314, 20.9%), followed by gastric cancer (*n*=203), and colon cancers (*n*=170). For mortality, lung cancer was the commonest cause of cancer death in men (*n*=167, 22.0%), followed by gastric (*n*=148) and liver cancer (*n*=74); in women, gastric cancer was the commonest cause of cancer death (*n*=58, 12.9%), followed by lung (*n*=56), and breast cancer (*n*=40).

At baseline, 70% of men were regular drinkers and 48% consumed alcohol 3–4 times per week or more; in women, 77% were nondrinkers and 12% regular drinkers. The average frequency of alcohol consumption among regular drinkers was 5.2 days per week in men and 3.6 days per week in women. Both in men and women (Table 1), the proportion of current smokers was increased in the higher ethanol intake groups, in which leisure-time physical activities were less frequent. However, no consistent trend in green vegetable intake was observed across the alcohol categories.

The adjusted cancer HRs by alcohol category are presented in Table 2, together with the ratios for cancer mortality. In men, the lowest risk was observed in occasional drinkers, compared to whom a significant increase in risks of cancer occurrence was observed as ethanol intake increased among regular drinkers (1–149 g per week: HR=1.18, 150–299 g per week: HR=1.17, 300–449 g per week: HR=1.43, ⩾450 g per week: HR=1.61, *P* for trend <0.001). This trend did not change when cases where the cancers occurred within the first five years of the study were excluded. For total cancer mortality, a similar trend was observed. Among males, 12.5% of the cancers in the study period were attributable to heavy drinking (⩾300 g of ethanol per week): 5.4% for the consumption of 300–449 g of ethanol per week and 7.1% for the consumption of ⩾450 g per week. Similar results were obtained for cancer mortality. Unlike men, cancer risk was the highest among occasional drinkers in women, but none of the risk values reached statistical significance.

The HRs for cancer in men were estimated separately by smoking status at baseline for each alcohol-drinking category (Table 3). For cancer incidence, no risk fluctuation was observed among never-smokers, whereas current smokers exhibited a constantly elevated risk compared with occasional drinkers (1–149 g per week: HR=1.69, 150–299 g per week: HR=1.64, 300–449 g per week: HR=1.93, ⩾450 g per week: HR=2.32, *P* for trend <0.001). Similar trends were observed for cancer mortality, with a somewhat decreased risk tendency among never-smokers. A statistically significant interaction between alcohol and smoking status applied to the risks of both total cancer incidence (*P*=0.018) and mortality (*P*<0.001).

The HRs for cancer in men were also separately determined for alcohol-related and other cancers (Table 3). As expected, increased risks were more evident for alcohol-related cancers than those for other cancers. In a further analysis restricted to alcohol-related cancers by smoking status, increased risks for the high-alcohol categories were also observed among never smokers, though less than among current smokers.

## DISCUSSION

In this cohort study, the lowest risk of cancer was observed among male occasional drinkers, and a linear positive association with increasing ethanol intake was seen, with up to a 61% excess cancer risk among subjects with an ethanol intake of ⩾450 g. The positive association was similar for both the cancer incidence and mortality, but was more striking among current smokers and for alcohol-related cancers. Considerable interaction between smoking and alcohol drinking was observed. On the other hand, no clear association between alcohol drinking and cancer was found in women, probably because few of them were regular drinkers. Among males, nearly 13% of the cancers were considered attributable to heavy drinking (⩾300 g of ethanol per week).

We assigned occasional drinkers to the reference category since nondrinkers were a mixture of never- and ex-drinkers, both of which contained subjects who are unable to drink due to a deficiency in the key enzyme for alcohol metabolism, common in the Japanese population. This might have complicated the interpretation of the results for nondrinkers. We were unable to assess the risk of never- and ex-drinkers separately, since the baseline questionnaire for cohort I did not discriminate abstainers from nondrinkers. Additional analyses using cohort II subjects, however, found that only 2% of all male subjects were ex-drinkers, and also no marked difference in the association among nondrinkers whether ex-drinkers were included or excluded. If this was also the case in cohort I, it seems unlikely that ‘never-drinking’ would be associated with an increased risk of cancer, though the possible residual confounding effects cannot be ruled out. Approximately half of the Japanese individuals were found to have a deficient phenotype for aldehyde dehydrogenase-2, a key enzyme in the conversion of acetaldehyde to acetate (Agarwal *et al*, 1981; Shibuya and Yoshida, 1988), resulting in higher levels of acetaldehyde exposure, which is considered to be carcinogenic (IARC, 1988). The fraction of cancer risk attributable to alcohol might therefore be greater among Japanese than among Western drinkers.

Previous studies have indicated that the effect of alcohol drinking does not appear to be due to any specific type of alcoholic beverage, but rather due to ethanol itself. In our study, most male regular drinkers drank two or more types of alcohol; 36% of the total ethanol intake among these subjects was from Japanese sake (rice wine), 33% from Japanese hard liquor, 24% from beer and 7% from whisky, whereas <1% was from wine and other alcoholic beverages.

Our results indicate that the combination of alcohol drinking and smoking is associated with a particularly increased risk of cancer and presumably makes a major contribution to both incidence and mortality of the overall cancer risk, while no such tendency was detected among never-smokers. Except for our previous work, studies of the effect of interaction between drinking and smoking on the total cancer risk in the Japanese are sparse (Tsugane *et al*, 1999; Hara *et al*, 2002). CYP2E1, the expression of which is induced by alcohol, metabolises procarcinogens, such as *N*-nitroso compounds, present in tobacco smoke and foods (Anderson *et al*, 1994); it also catalyses the conversion of alcohol to acetaldehyde. Animal experiments have suggested that carcinogens in tobacco smoke are metabolised more slowly among drinkers (Van de Wiel *et al*, 1993; Anderson *et al*, 1994). Although interaction between alcohol and smoking may greatly contribute to the risk of both cancer incidence and mortality, alcohol may also be an independent risk factor, at least for alcohol-related cancers.

The major strengths of our study were its prospective design, its high response rate, and the negligible proportion of losses to follow-up. The collection of alcohol details before cancer diagnosis precluded the exposure recall bias inherent in case–control studies. However, misclassification of the self-reported alcohol due to modified alcohol-drinking behaviour during the study period is possible. However, these would probably be nondifferential and may underestimate the true relative risk. Although the quality of the cancer registry system was satisfactory during the study period, there was some geographical variation by study area, so this was adjusted for in the analysis. We also confirmed that the quality of the registry was not affected by drinking status at baseline, so underreporting of cancer should also have been nondifferential.

Our cohort study found that an increased ethanol intake substantially elevates the risk of total cancer, but this effect appeared to be largely due to interactions with smoking. For cancer prevention therefore, combined cessation of smoking and alcohol drinking is important for the reduction of cancer risk.

## Figures and Tables

**Table 1:** Baseline characteristics of the study subjects according to alcohol drinking category

		**Alcohol drinking category**					
				**Weekly ethanol intake (g per week)**			
	**Total**	**Nondrinkers**	**Occasional drinkers**	**1–149**	**150–299**	**300–449**	**⩾450**
*Men (n=35 007)*							
Number of subjects	35 007	7009	3555	7853	7039	4899	4652
Proportion (%)		20.0	10.2	22.4	20.1	14.0	13.3
Age (years) ±s.d.	49.1±5.9	49.8±6.0	48.6±5.9	48.7±6.0	49.0±5.9	49.4±5.8	49.1±5.9
*Smoking status (%)*							
Never	24.7	29.5	33.0	30.2	21.3	16.9	15.5
Former	21.1	19.8	19.0	22.7	21.9	21.6	20.1
Current	54.2	50.7	48.0	47.1	56.8	61.5	64.4
*Green vegetable intake (%)*							
Almost everyday	23.5	23.8	24.1	22.4	23.2	24.4	23.6
*Leisure-time physical activity (%)*							
⩾ 1–2 times per week	18.0	15.2	18.7	21.0	18.5	17.9	15.6
*Women (n=38 274)*							
Number of subjects	38 274	29 356	4329	3584	593	191	221
Proportion (%)		76.7	11.3	9.4	1.5	0.5	0.6
Age (years) ±s.d.	49.4±5.9	49.8±5.9	47.8±5.7	48.0±5.6	47.9±5.7	47.2±5.4	47.3±5.5
*Smoking status (%)*							
Never	92.4	94.8	90.4	85.6	60.6	52.9	49.3
Former	1.4	1.0	2.1	2.4	6.3	6.8	3.6
Current	6.2	4.2	7.5	12.0	33.2	40.3	47.1
*Green vegetable intake (%)*							
Almost everyday	31.1	31.6	28.2	31.1	27.9	22.2	27.9
*Leisure-time physical activity (%)*							
⩾1–2 times per week	15.8	15.0	18.1	19.3	16.9	14.7	13.2

**Table 2:** Hazard ratios (HR)^a^ and 95% confidence interval (95% CI) of cancer incidence and deaths according to alcohol-drinking status

				**Weekly ethanol intake (g per week)**				
		**Nondrinkers**	**Occasional drinkers**	**1–149**	**150–299**	**300–449**	**⩾450**	***P* for trend**
**Men (*n*=35 007)**								
*Cancer incidence*	Person-years	68 161.0	35 151.9	74 007.4	67 934.7	48 173.5	46 194.9	
Total (*n=*1904)								
No. of cases		360	138	353	359	339	355	
HR (95% CI)		1.10 (0.90–1.34)	1.00 (reference)	1.18 (0.96–1.44)	1.17 (0.96–1.44)	1.43 (1.17–1.75)	1.61 (1.32–1.97)	<0.001
PAF% (95% CI)						5.4 (1.4–9.1)	7.1 (2.9–11.0)	
Excluding first 5 years (*n=*1172)								
No. of cases		214	81	217	234	206	220	
HR (95% CI)		1.11 (0.86–1.44)	1.00 (reference)	1.25 (0.97–1.62)	1.29 (0.99–1.67)	1.44 (1.10–1.87)	1.64 (1.26–2.12)	0.002
PAF% (95% CI)						5.4 (0.3–10.2)	7.3 (1.9–12.4)	
								
*Cancer deaths*	Person-years	69 175.7	35 547.7	75 042.2	69 039.4	49 194.1	47 259.3	
Total (*n=*758)								
No. of cases		161	59	138	119	133	148	
HR (95% CI)		1.10 (0.81–1.49)	1.00 (reference)	1.06 0.77–1.44)	0.92 (0.67–1.26)	1.33 (0.97–1.83)	1.58 (1.16–2.15)	<0.001
PAF% (95% CI)						4.4 (−1.7–10.1)	7.1 (0.4–13.4)	
Excluding first 5 years (*n=*533)								
No. of cases		107	35	106	82	96	107	
HR (95% CI)		1.25 (0.85–1.85)	1.00 (reference)	1.39 (0.94–2.06)	1.08 (0.72–1.62)	1.62 (1.09–2.42)	1.90 (1.28–2.81)	0.009
PAF% (95% CI)						6.9 (−1.1–14.3)	9.5 (0.6–17.6)	
								
**Women (*n*=38 274)**								
*Cancer incidence*	Person-years	293 866.0	43 116.9	34 966.7	5757.5	1803.8	2168.2	
Total (*n=*1499)								
No. of cases		1170	178	118	20	6	7	
HR (95% CI)		0.94 (0.80–1.11)	1.00 (reference)	0.80 (0.63–1.01)	0.68 (0.42–1.11)	0.73 (0.32–1.66)	0.68 (0.32–1.46)	0.659
Excluding first 5 years (*n=*908)								
No. of cases		707	115	68	12	3	3	
HR (95% CI)		0.88 (0.72–1.08)	1.00 (reference)	0.70 (0.51–0.95)	0.59 (0.31–1.13)	0.59 (0.19–1.88)	0.47 (0.15–1.49)	0.351
								
*Cancer deaths*	Person-years	298 259.9	43 775.0	35 434.0	5827.6	1821.6	2187.6	
Total (*n=*450)								
No. of cases		368	43	28	6	3	2	
HR (95% CI)		1.08 (0.79–1.49)	1.00 (reference)	0.79 (0.49–1.27)	0.54 (0.19–1.52)	1.27 (0.39–4.15)	0.68 (0.16–2.86)	0.896
Excluding first 5 years (*n=*325)								
No. of cases		273	31	17	3	1	0	
HR (95% CI)		1.14 (0.78–1.66)	1.00 (reference)	0.69 (0.38–1.25)	0.20 (0.03–1.51)	0.69 (0.09–5.09)	—	0.289

a Adjusted for age at baseline (continuous), study area (9 PHC area), pack-years of smoking (0, 1–19, 20–29, 30–39, ⩾40), green vegetable intake (⩽3–4 times per week, almost everyday), and leisure-time physical activity (⩽1–3 times per month, ⩾1–2 times per week).

**Table 3:** Hazard ratios (HR)^a^ and 95% confidence interval (95% CI) of cancer incidence and deaths attributed to alcohol-related cancers and nonalcohol-related cancers^b^ according to the alcohol drinking status in men (*n=*35 007)

			**Weekly ethanol intake (g per week)**					
	**Nondrinkers**	**Occasional drinkers**	**1–149**	**150–299**	**300–449**	**⩾450**	***P* for trend**	***P* for interaction**
*Cancer incidence*								
* Never-smokers (n=315)*								
No. of cases	78	42	75	54	37	30		
HR (95% CI)	0.90 (0.62–1.31)	1.00 (reference)	0.87 (0.60–1.28)	0.86 (0.57–1.29)	1.03 (0.66–1.62)	1.02 (0.64–1.64)	0.370	
* Current smokers (n=1163)*								0.018
No. of cases	196	58	202	226	224	257		
HR (95% CI)	1.39 (1.03–1.88)	1.00 (reference)	1.69 (1.25–2.28)	1.64 (1.22–2.20)	1.93 (1.43–2.60)	2.32 (1.72–3.11)	<0.001	
								
**Alcohol–related cancers (*n=*250)**								
No. of cases	29	8	38	49	52	74		
HR (95% CI)	1.57 (0.72–3.45)	1.00 (reference)	2.28 (1.06–4.90)	2.95 (1.39–6.28)	4.03 (1.90–8.56)	6.16 (2.95–12.8)	<0.001	
*Never smokers (n=34)*								
No. of cases	7	2	8	5	6	6		
HR (95% CI)	1.88 (0.39–9.09)	1.00 (reference)	2.09 (0.44–9.95)	1.91 (0.37–9.95)	3.42 (0.65–17.9)	4.70 (0.93–23.7)	0.109	
*Current smokers (n=163)*								
No. of cases	14	5	19	36	33	56		
HR (95% CI)	1.12 (0.40–3.15)	1.00 (reference)	1.95 (0.73–5.24)	3.23 (1.26–8.28)	3.72 (1.44–9.62)	6.41 (2.55–16.1)	<0.001	
								
**Nonalcohol-related cancers (*n=*1654)**								
No. of cases	331	130	315	310	287	281		
HR (95% CI)	1.07 (0.87–1.31)	1.00 (reference)	1.11 (0.90–1.36)	1.06 (0.86–1.31)	1.27 (1.03–1.57)	1.34 (1.11–1.66)	0.004	
*Never smokers (n=282)*								
No. of cases	71	40	67	49	31	24		
HR (95% CI)	0.85 (0.57–1.26)	1.00 (reference)	0.81 (0.55–1.20)	0.80 (0.53–1.23)	0.92 (0.57–1.48)	0.85 (0.51–1.42)	0.733	
*Current smokers (n=1000)*								
No. of cases	182	53	183	190	191	201		
HR (95% CI)	1.41 (1.03–1.93)	1.00 (reference)	1.66 (1.21–2.27)	1.48 (1.08–2.03)	1.76 (1.28–2.41)	1.94 (1.42–2.66)	0.039	
								
*Cancer deaths*								
* Never–smokers (n=124)*								
No. of cases	36	25	27	19	7	10		
HR (95% CI)	0.67 (0.40–1.12)	1.00 (reference)	0.53 (0.31–0.92)	0.49 (0.27–0.91)	0.33 (0.14–0.78)	0.55 (0.26–1.16)	0.634	
* Current smokers (n=484)*								<0.001
No. of cases	81	23	83	84	99	114		
HR (95% CI)	1.43 (0.89–2.31)	1.00 (reference)	1.68 (1.04–2.69)	1.52 (0.94–2.44)	2.15 (1.35–3.44)	2.57 (1.62–4.09)	<0.001	
								
**Alcohol–related cancers (*n=*143)**								
No. of cases	19	5	25	28	31	35		
HR (95% CI)	1.58 (0.59–4.25)	1.00 (reference)	2.26 (0.86–5.92)	2.55 (0.98–6.67)	3.86 (1.48–10.0)	4.89 (1.90–12.5)	0.003	
*Never smokers (n=22)*								
No. of cases	7	2	4	4	2	3		
HR (95% CI)	1.76 (0.36–8.54)	1.00 (reference)	1.01 (0.18–5.59)	1.45 (0.26–8.04)	1.29 (0.18–9.37)	2.35 (0.38–14.4)	0.280	
*Current smokers (n=89)*								
No. of cases	6	3	14	20	20	26		
HR (95% CI)	0.81 (0.20–3.26)	1.00 (reference)	2.17 (0.62–7.57)	2.66 (0.78–9.07)	3.45 (1.01–11.8)	4.78 (1.43–15.9)	0.014	
								
**Nonalcohol-related cancers (*n=*615)**								
No. of cases	142	54	113	91	102	113		
HR (95% CI)	1.06 (0.77–1.45)	1.00 (reference)	0.94 (0.68–1.31)	0.76 (0.54–1.08)	1.09 (0.78–1.53)	1.27 (0.91–1.77)	0.010	
*Never smokers (n=102)*								
No. of cases	29	23	23	15	5	7		
HR (95% CI)	0.57 (0.33–1.00)	1.00 (reference)	0.49 (0.27–0.88)	0.41 (0.21–0.80)	0.26 (0.10–0.68)	0.41 (0.17–0.96)	0.287	
*Current smokers (n=395)*								
No. of cases	75	20	69	64	79	88		
HR (95% CI)	1.53 (0.92–2.54)	1.00 (reference)	1.60 (0.96–2.67)	1.34 (0.80–2.25)	1.96 (1.18–3.25)	2.25 (1.36–3.72)	0.008	

a Adjusted for age at baseline (continuous), study area (9 PHC area), daily cigarette consumption (number of cigarettes, continuous, current smoker only), green vegetable intake (⩽3–4 times per week, almost everyday), and leisure-time physical activity (⩽1–3 times per month, ⩾1–2 times per week).

b Alcohol-related cancers consist of cancer of the oral cavity, pharynx, oesophagus, liver, and larynx. Nonalcohol-related cancers consist of all other cancers not considered to be alcohol related.

## Appendix

Members of the Japan Public Health Center-based Prospective Cohort Study on Cancer and Cardiovascular Diseases (JPHC Study) Group: S Tsugane, T Sobue, T Hanaoka, M Inoue, National Cancer Center; J Ogata, S Baba, T Mannami, A Okayama, National Cardiovascular Center; K Miyakawa, F Saito, A Koizumi, Y Sano, I Hashimoto, Iwate Prefectural Ninohe Public Health Center; Y Miyajima, N Suzuki, S Nagasawa, Y Furusugi, Akita Prefectural Yokote Public Health Center; H Sanada, Y Hatayama, F Kobayashi, H Uchino, Y Shirai, T Kondo, R Sasaki, Y Watanabe, Nagano Prefectural Saku Public Health Center; Y Kishimoto, E Tanaka, M Kinjo, T Fukuyama, M Irei, Okinawa Prefectural Chubu Public Health Center; K Imoto, H Yazawa, T Seo, A Seiko, F Ito, Katsushika Public Health Center; A Murata, K Minato, K Motegi, T Fujieda, Ibaraki Prefectural Mito Public Health Center; K Matsui, T Abe, M Kataoka, Niigata Prefectural Kashiwazaki Public Health Center; M Doi, Y Ishikawa, A Terao, Kochi Prefectural Chuo-higashi Public Health Center; H Sueta, H Doi, M Urata, Nagasaki Prefectural Kamigoto Public Health Center; H Sakiyama, N Onga, H Takaesu, Okinawa Prefectural Miyako Public Health Center; F Horii, I Asano, H Yamaguchi, K Aoki, S Maruyama, M Ichii, Osaka Prefectural Suita Public Health Center; S Matsushima, S Natsukawa, Saku General Hospital; S Watanabe, M Akabane, Tokyo University of Agriculture; M Konishi, K Okada, Ehime University; H Iso, Y Honda, Tsukuba University; H Sugimura, Hamamatsu University School of Medicine; Y Tsubono, Tohoku University; T Kadowaki, Tokyo University; N Kabuto, National Institute for Environmental Studies; S Tominaga, Aichi Cancer Center; M Iida, W Ajiki, Osaka Medical Center for Cancer and Cardiovascular Disease; S Sato, Osaka Medical Center for Health Science and Promotion; N Yasuda, Kochi Medical School; S Kono, Kyushu University; K Suzuki, Research Institute for Brain and Blood Vessels Akita; Y Takashima, Kyorin University; E Maruyama, Kobe University; M Yamaguchi, Y Matsumura, S Sasaki, National Institute of Health and Nutrition.
